# Dynamics of West Nile Virus Persistence in House Sparrows (*Passer domesticus*)

**DOI:** 10.1371/journal.pntd.0001860

**Published:** 2012-10-04

**Authors:** Sarah S. Wheeler, Meighan P. Vineyard, Leslie W. Woods, William K. Reisen

**Affiliations:** 1 Center for Vectorborne Diseases, Department of Pathology, Microbiology and Immunology, School of Veterinary Medicine, University of California Davis, Davis, California, United States of America; 2 California Animal Health and Food Safety, Department of Pathology, Microbiology and Immunology, School of Veterinary Medicine, University of California Davis, Davis, California, United States of America; University of Texas Medical Branch, United States of America

## Abstract

West Nile Virus (WNV) is now endemic throughout North America, with annual recurrence dependent upon successful overwintering when cold temperatures drive mosquito vectors into inactivity and halt transmission. To investigate whether avian hosts may serve as an overwintering mechanism, groups of eight to ten House Sparrows were experimentally infected with a WN02 genotype of WNV and then held until necropsy at 3, 5, 7, 9, 12, 15, or 18 weeks post-infection (pi) when they were assessed for the presence of persistent infection. Blood was collected from all remaining birds every two weeks pi, and sera tested for WNV RNA and WNV neutralizing antibodies. West Nile virus RNA was present in the sera of some birds up to 7 weeks pi and all birds retained neutralizing antibodies throughout the experiment. The detection of persistently infected birds decreased with time, from 100% (n = 13) positive at 3 weeks post-infection (pi) to 12.5% (n = 8) at 18 weeks pi. Infectious virus was isolated from the spleens of birds necropsied at 3, 5, 7 and 12 weeks pi. The current study confirmed previous reports of infectious WNV persistence in avian hosts, and further characterized the temporal nature of these infections. Although these persistent infections supported the hypothesis that infected birds may serve as an overwintering mechanism, mosquito-infectious recrudescent viremias have yet to be demonstrated thereby providing proof of principle.

## Author Summary

House Sparrows experimentally infected with West Nile virus [WNV] were necropsied at multiple time points from 3 to 18 weeks post infection (pi). The percent of birds with tissues positive for WNV RNA decreased from 100% at 3 wks to 13% at 18 wks pi; infectious virus was recovered from some birds by tissue co-cultivation and Vero cell passage from 3 to 12 wks pi, even though positive birds retained neutralizing antibody. WNV RNA also was detected in sera at 2 to 7 wks pi. Collectively, these data indicated that House Sparrows frequently developed persistent infections and could serve as an overwintering mechanism for WNV. However, recrudescent viremias suitable to infect mosquitoes have yet to be demonstrated and would seem to require host Immunosuppression.

## Introduction

West Nile virus (WNV; *Flaviviridae: Flavivirus*) is a relatively new arbovirus in the Western Hemisphere that has been detected each year [1] since its introduction into New York, NY in 1999. West Nile virus is an enveloped, single-stranded RNA virus [2] that is primarily transmitted in an enzootic cycle involving *Culex* mosquitoes and passerine birds. Humans and horses are infected tangentially and generally do not contribute to the transmission cycle. The success of the WNV invasion can be attributed, in part, to the presence of competent mosquito vectors and avian hosts [3]–[5], and to the virus' ability to survive temperate winters that drive mosquito vectors into inactivity and halt the transmission cycle.

The mechanisms allowing WNV to overwinter likely rely on persistent infection of either mosquito vectors or avian hosts. Previous studies have reported the winter collection of WNV-infected *Culex* mosquitoes [6]–[9]. Vertical transmission of WNV in mosquitoes, although demonstrated infrequently [10]–[13], was most likely the mechanism by which these overwintering mosquitoes became infected.

Alternatively, persistent WNV infections have been described in vertebrates, including mice (*Mus musculus*) [14] and Golden Hamsters (*Mesocricetus auratus*) [15]. However, because these rodents are not a natural host for WNV, direct implications for WNV overwintering cannot be inferred. Persistent WNV infections also have been reported in several avian species. Early work described the recrudescence of WNV persistent infections in Blue-gray Pigeons (*Columba* cf. *livia*), with virus isolated from blood at 16, 93 and 100 days post-infection (pi) [16]. Reisen et al. [13] demonstrated that WNV RNA could be demonstrated in spleen, kidney and lung tissues in several avian species up to 6 weeks pi. In addition, infectious virus was isolated from four of six of RNA positive House Finches (*Carpodacus mexicanus*) after passage in C6/36 mosquito cells. Nemeth et al. [17] isolated infectious virus from the mouth of an experimentally infected House Sparrow (*Passer domesticus*) 44 days pi and found RNA in kidney and/or spleen tissues at 65 days pi. Our previous work [18] revealed that WNV RNA persisted in the spleen and/or kidney tissues of some experimentally infected birds up to 6 months pi and in some naturally-infected birds greater than 4 months pi. Because attempts at isolating infectious virus at these time periods failed [18], it is unknown whether this detection of WNV RNA could be attributed to infectious virus, with the potential to recrudesce and restart transmission.

The purpose of the current work was to characterize temporal changes in persistent WNV infections using the House Sparrow as an avian model. This was accomplished by conducting a time course experiment in which House Sparrows were experimentally infected with WNV, then evaluated for viral persistence at multiple time points up to 18 weeks pi. Spleen, kidney, skin, and brain tissues taken at necropsy were tested for both WNV RNA and infectious virus. In addition, birds were monitored throughout the experiment for recrudescence by screening sera for WNV RNA and for immune status by testing for neutralizing antibodies.

## Materials and Methods

### Ethics

This study was performed in strict accordance with the recommendations in the Guide for the Care and Use of Laboratory Animals of the National Institutes of Health. The University of California Davis campus is approved for animal studies by the US National Institutes of Health under Animal Welfare Assurance number A3433. The collection, housing, transport, infection and euthanasia of birds were conducted under approved University of California, Davis, Institutional Animal Care and Use Committee protocols 12876 and 12880. Birds were collected by grain-baited traps and mist nets under USGS Master Station Banding Permit 22763 and State of California Scientific Collecting Permit 801281-01 and taken for experimentation under Federal Permit MB082812. BSL3 laboratory facilities were approved under Biological Use Authorization 0554 and 0873 by the University of California, Davis, Environmental Health and Safety Institutional Biosafety Committee and USDA Permit 47901.

### Cells and Virus

African Green Monkey kidney (Vero) cells were maintained in Dulbecco's Modified Eagle medium (DMEM; Life Technologies: Gibco, Carlsbad, CA) supplemented with 10% fetal bovine serum (FBS), 500 U/mL penicillin and 0.5 mg/mL streptomycin, and maintained at 37°C and 5% C0_2_. The WNV strain used was isolated originally from a dead Yellow-billed Magpie (*Pica nuttalli*) collected in 2004 in Sacramento, CA (WNV CA04, GenBank accession number DQ080059), and had been passaged three times on Vero cells prior to experimentation. Viral titers were assessed by Vero cell plaque assay [19].

### Birds

House Sparrows were selected as an avian model because they were considered an important maintenance host [3], [4] for WNV, and frequently have been reported positive for WNV antibodies in seroprevalence studies [20], [21]. In addition, House Sparrows were abundant, easily collected, adapted well to laboratory conditions, and previously were shown to develop persistent WNV infections [13], [17], [18]. Birds were collected in Kern and Yolo Counties, CA, banded with a uniquely numbered aluminum leg band, screened for antibodies attributable to previous WNV infection, and given a minimum of two weeks for cage-adaption. During the cage-adaption period, a 14-day course of chlorotetracycline (Fort Dodge; Overland Park, KS) was administered in drinking water at 0.2 mg/mL. Until adequate numbers were collected, birds were held at the Arbovirus Field Station in Bakersfield, CA or the University of California, Davis, in mosquito-proof, outdoor flight cages equipped with perches, water and food dispensers, and bird baths. Prior to WNV infection, birds were transported to the California Animal Health and Food Safety Laboratory (CAHFS) animal containment facility in Davis, CA where they were held in wire cages [dimensions: 0.9 m(W)×0.6 m(L)×0.6 m(H)] within Horsfall-Bauer containment units, each fitted with a HEPA-filtered negative air system. Similar to outdoor aviaries, cages were furnished with perches, water baths, and food and water dispensers. Diet included a mixture of formulated High Energy Breeder Diet (Roudybush™; Woodland, CA) and mixed bird seed including: canary grass seed, oat chips, golden German millet, canola rape seed, flax seed, Nyjer seed and hemp seed (The Seed Factory; Ceres, CA). Sparrows were assigned to cages and study groups, with seven or less individuals per cage. Birds were needle-inoculated with 10^3^ plaque forming units (pfu) of WNV, suspended in 0.05 mL viral transport diluent (DMEM, 500 U/mL penicillin, 0.5 mg/mL streptomycin and 20% FBS), subcutaneously over the upper right pectoral muscle. Negative controls were sham-inoculated with transport diluent alone. Blood was collected in 0.1 mL volumes by jugular venipuncture and diluted in 0.45 mL of transport diluent, for a 1∶10 serum dilution. Clinical signs of WNV infection were rarely observed, but included puffing of feathers, lethargy, and failure to react when disturbed. Birds were euthanized prior to necropsy by CO_2_ inhalation.

### Study Design

**Figure 1:**
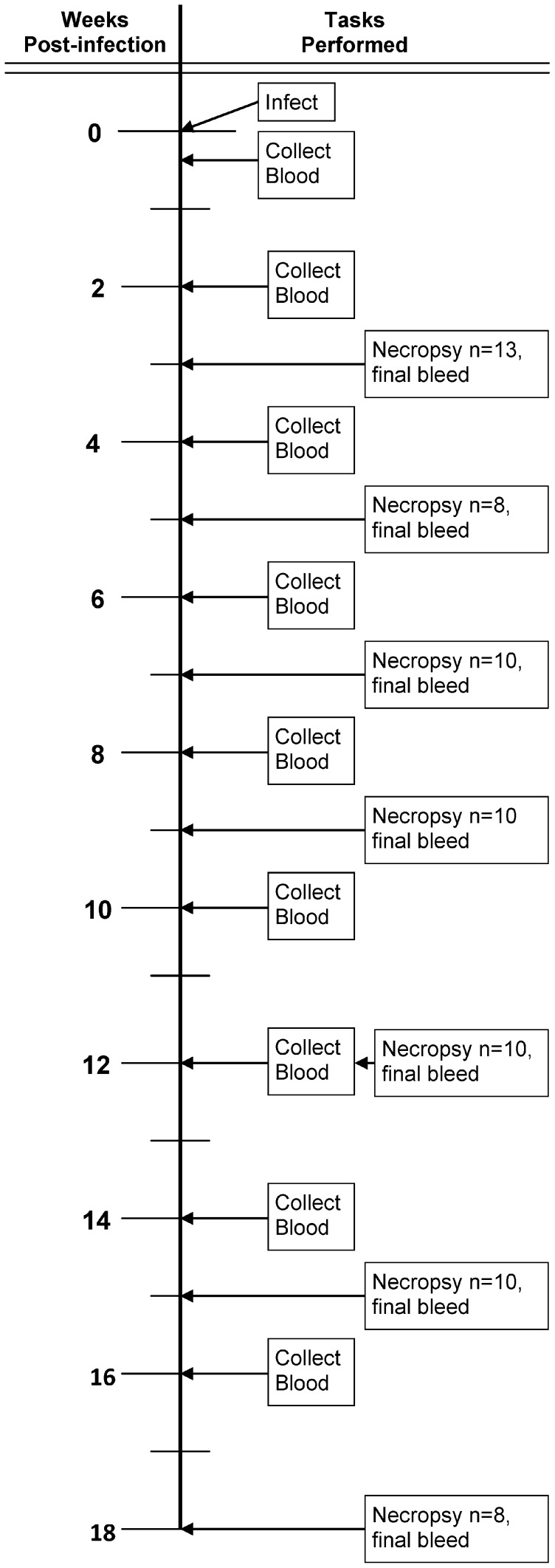
Experimental design showing the chronology of sampling events.

Groups of 8 to 12 experimentally infected House Sparrows were euthanized at 3, 5, 7, 9, 12, 15 and 18 weeks post-infection. Negative controls were sacrificed concurrently with the last time point. Blood samples were collected at three or four days pi to measure the viremia response and then biweekly throughout the experiment (Figure 1). After blood collection all samples were centrifuged (9000× g; 5 min), sera collected, aliquoted, and either heat-inactivated and stored at −20°C for serology, or stored at −80°C for RNA extraction and detection by qRT-PCR. At the end of each holding period, a final blood sample was collected, House Sparrows were euthanized by CO_2_ inhalation, and tissues were collected to assess WNV infection.

### Tissue Harvesting and Processing

At necropsy, spleen, kidney, brain including a portion of the cerebellum, and skin from over the pectoral muscle at the inoculation site were collected. To prevent cross-contamination tools were cleaned between birds using 1-Stroke Eviron Germicidal Detergent (Steris; Mentor, OH). After collection, each tissue was placed into cryovials and kept cold on wet ice until processing; tissues were not allowed to freeze. Co-culture medium (DMEM, 500 U/mL penicillin, 0.5 mg/mL streptomycin, 0.05 mg/mL gentamicin sulfate, 2 µg/mL amphotericin B solution, and 10% FBS) was added to each sample to make a 2.5% tissue homogenate. Two 5 mm glass beads were added to each tissue sample, whereas two 5 mm copper ball-bearings were added to skin samples. Tissues were homogenized by mixer mill (MM300, Retsch; Haan, Germany) at a frequency of 24 cycles/second for four min.

### Assays

All sera were tested for antibodies by plaque reduction neutralization test [22], using a 90% end point (PRNT_90_). Serum samples were heat-inactivated at 56°C for 30 min, and serially diluted 2-fold starting at 1∶10. Diluted sera were mixed 1∶1 with virus diluent containing approximately 100 pfu of WNV (CA04). Assays were conducted using confluent Vero cells grown in 6-well plates, with 0.1 mL of virus/sera mixture added to the Vero cell monolayer and allowed to incubate for one hour at 37°C, 5% CO_2_. A double overlay system was used; the first overlay (nutrient medium, 1% agarose and 2.3 mg/mL sodium bicarbonate) was applied after the incubation period. The second overlay (nutrient medium, 1% agarose, 0.1 mg/mL neutral red and 2.3 mg/mL sodium bicarbonate) was applied 48 h after the first; plates were read 72 h after inoculation. The highest dilution at which >90% of >75 pfu were neutralized was considered the PRNT_90_ end point titer.

Sera collected on days three and four were tested for infectious virus by plaque assay [19] using Vero cell cultures in 6-well plates. Sera were serially diluted 10-fold and allowed to incubate for 60 min at 37°C with 5% C0_2_. Overlay schedule was as explained above. Plaques were enumerated at 72 h to obtain viral titers. Because sera were diluted 1∶10 at collection, the detection limit was 100 pfu/mL.

### RNA Extraction

Total RNA was extracted from tissue homogenates the day of necropsy; sera were frozen at −80°C and RNA extracted within four months of collection. RNA was extracted from a 50 µL sample of tissue homogenate or sera using a MagMAX™ 96 and viral isolation kits according to manufacturer protocols (Life Technologies: Applied Biosystems; Carlsbad, CA, USA). Care was taken to avoid cross-contamination and numerous negative control wells containing only viral transport medium were included on each extraction plate. Confirmation samples from virus isolation attempts were processed on a different day. Contamination was not observed throughout in tissue homogenates, sera from sham-inoculated sparrows, or in negative control wells containing viral transport medium alone.

### qRT-PCR

RNA samples were analyzed for the presence and quantity of WNV RNA by TaqMan one-step quantitative reverse transcriptase-polymerase chain reaction (qRT-PCR) utilizing an ABI7900 platform (Life Technologies; Applied Biosystems; Carlsbad, CA, USA). Samples were evaluated with two primer/probe sets in separate reactions. The first set, WN1, was specific for the envelope region of the viral genome (WN1) [23]: (forward) 5′- TCA GCG ATC TCT CCA CCA AAG -3′, (reverse) 5′- GGG TCA GCA CGT TTG TCA TTG -3′, and (probe) 6FAM-TGC CCG ACC ATG GGA GAA GCT –TAMRA. Confirmation was attempted with a second primer/probe set (WN2) specific for NS1 region of the viral genome [24]: (forward) 5′-GGC AGT TCT GGG TGA AGT CAA -3′, (reverse) 5′-CTC CGA TTG TGA TTG CCT CGT -3′, and (probe) 6FAM-TGT ACG TGG CCT GAG ACG CAT ACC TTG T-TAMRA. All samples with a threshold cycle (C_t_) score <40 were considered WN1 and/or WN2-positive. Sera with WN1 C_t_ scores >30 that failed to confirm with WN2 were re-extracted and qRT-PCR was repeated using the WN1 primers/probe. All sample plates contained a standard curve generated from cultured virus of known titer (plaque forming units/mL) and negative water controls.

### Virus Isolation

Tissue homogenates were evaluated for infectious virus using a co-culture method adapted from Appler et al. [14] and others [15], [25]. In brief, Vero cells were grown in co-culture medium, as described above, to 75% confluence in 6-well plates; 100 µL of tissue homogenate was added to each well. Plates were observed daily for cytopathic effect (CPE; rolling of cells and/or sloughing of the monolayer); when CPE was observed the cell culture supernatant was tested for WNV antigen by VecTest (Medical Analysis Systems Inc., Camarillo, CA). If WNV antigen was detected, the supernatant was collected and no further passages were attempted. After seven days, 1.0 mL of supernatant was transferred onto fresh Vero cells. Each homogenate was passaged three times, unless WNV antigen was detected. After three passages RNA was extracted from the CPE negative samples and tested for WNV RNA by qRT-PCR.

### Statistics

Statistical analyses were performed using GraphPad Prism version 5.04 for Windows (GraphPad Software; La Jolla, CA). Student's *t* tests compared mean viremia titers (log_10_ pfu/mL) between birds bled on either three or four days pi, and between birds that survived or succumbed to WNV infection. Student's *t* test was also used to compare mean WN1 qRT-PCR C_t_ scores between samples that were WN2 primer/probe confirmed and unconfirmed. To test whether WNV persistence as indicated by recovery of RNA at necropsy led to greater antibody titers, log_e_ transformed PRNT_90_ antibody titers were compared by a 2-way general linear model ANOVA with persistence status and time after infection as main effects.

## Results

### Viremia and Antibody Responses

Overall, 85 House Sparrows were infected experimentally with WNV, and 6 were sham-inoculated and held as negative controls. Over the course of the experiment, two birds died after blood sampling (one of which was a negative control) and two died approximately three weeks post-infection of unknown causes. In total, 13 birds succumbed during acute WNV infection between days two and twelve pi, with the majority (54%) succumbing on the sixth day.

To decrease stress birds were bled only once during the acute infection period. Based on our previous studies and the literature, blood was collected at four days pi to measure the magnitude of peak viremia. Unexpectedly, 11 of 70 experimentally infected birds had sera that were negative for infectious virus by plaque assay at this time, but all of these sera were positive for WNV RNA by qRT-PCR. In addition, all developed a WNV-neutralizing antibody response. Therefore, blood was collected from the remaining birds (n = 13) at three dpi, at which time, all had detectable viremias by plaque-assay.

Viremia titers were transformed to log_10_ plaque forming units (pfu)/mL and compared among the birds that survived infection. The mean viremia (± standard deviation) of birds that survived acute infection and were bled on day three (4.2±1.1 log_10_ pfu/mL, n = 13) was significantly greater (*t* = 3.0, df = 58, P<0.005 two-tailed) than the mean viremia of surviving birds bled on day four (3.3±0.9 log_10_ pfu/mL, n = 47). Sample sizes for birds that succumbed to WNV infection were low. However, in contrast to surviving birds, there was no significant difference (*t* = 0.98, df = 8, P>0.05 two-tailed) in mean viremia titers between birds that succumbed to WNV infection and were bled at three (8.5±0.07 log_10_ pfu/mL, n = 2) or four dpi (7.2±1.8 log_10_ pfu/mL, n = 8). Birds that succumbed to infection had significantly higher viremias on both days three (*t* = 5.4, df = 13, *P*<0.001 two-tailed) and four pi (*t* = 9.4, df = 53, P<0.001 two-tailed) than those that survived.

**Figure 2:**
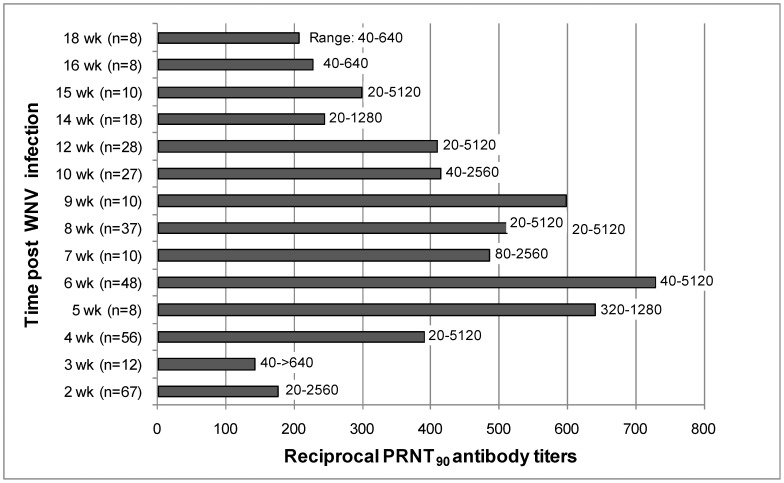
Geometric mean antibody titers for House Sparrows (*Passer domesticus*) experimentally infected with WNV. Titers were measured by a 90% plaque reduction neutralization assay, the sample size and range of the reciprocal titers is provided for each time point.

By 14 dpi all experimentally infected birds were PRNT_90_ positive for WNV antibodies, and as previously reported [26], the humoral response was robust. Figure 2 shows the inverse of the geometric mean titers at 2 to 18 weeks pi. Neutralizing antibody titers peaked between five and nine weeks pi, but all experimentally infected birds maintained titers ≥1∶20 throughout the experiment. None of the sham-inoculated birds developed a WNV-specific antibody response (PRNT_90_<1∶20). When necropsied at 5–18 weeks pi, the mean (± standard deviation) reciprocal of the log_e_ PRNT_90_ titer for birds with persistent RNA in one or more organs (6.53±0.98, back transformed mean = 645.5, n = 21) was not significantly greater (2-way ANOVA, F = 1.95, df = 1, 44, P = 0.17) than the mean for negative birds (5.79±1.35, back transformed mean = 358.9, n = 35). Similar results were obtained when titers from birds necropsied at weeks 5–9 pi were analyzed separately (data not shown, P>0.05).

### Viral Persistence

**Figure 3:**
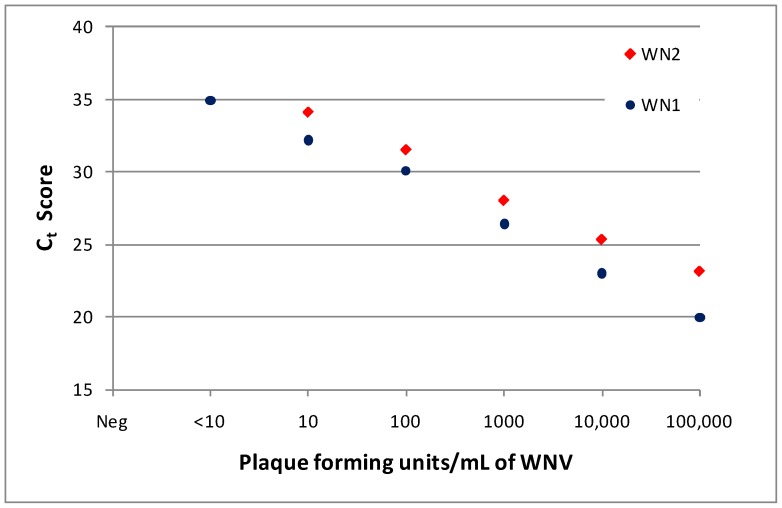
Relationship between threshold cycle (C_t_) score and West Nile virus (WNV) titer. Known titers of WNV (CA04 isolate) grown in Vero cell culture were analyzed with an Applied Biosystems 7900 Real-time PCR machine (see Methods section) utilizing WN1 and WN2 primer/probe sets, C_t_ scores are shown.

Sparrows were evaluated for persistent WNV at 3, 5, 7, 9, 12, 15, and 18 weeks pi. West Nile virus RNA was detected in the tissues of at least one bird at all time points, except for birds necropsied at 15 weeks pi. The number of birds in each group that were positive for WNV RNA decreased with time pi: 100% (n = 13) were positive at 3 weeks pi, 75% (n = 8) at 5 weeks pi, 50% (n = 10) at 7 and 9 weeks pi, 40% at 12 weeks pi, none (n = 10) at 15 weeks pi, and 12.5% (n = 8) at 18 weeks pi. In persistently infected birds, spleen and kidney tissues were most commonly RNA-positive; skin at the inoculation site and brain tissues were infrequently positive (Table 1). All birds were screened for WNV RNA by qRT-PCR using the WN1 primer/probe set. Tissue homogenate samples that were positive for WNV RNA (C_t_ score <40) were retested using the WN2 primer/probe set, and 25% (n = 4) of brain, 33% (n = 9) of skin, 75% (n = 20) of kidney, and 78% (n = 27) of spleen samples were again qRT-PCR positive (C_t_<40). The mean (± standard deviation) WN1 C_t_ score for tissue samples that confirmed by WN2 (27.6±2.8, n = 40) was significantly less (t = 6.3, df = 58, p = <0.001 one-tailed) than that of samples that failed to confirm (33.0±3.6, n = 20). These results are similar to previous findings [18], where the reduced sensitivity of WN2 primer/probe set limited confirmation of some samples with C_t_ scores >30. Figure 3 compares the sensitivity of WN1 and WN2 qRT-PCR assay results to WNV plaque assay titers for viral standards prepared in Vero cell culture.

**Table 1:** House Sparrows (*Passer domesticus*) tissues persistently infected with West Nile virus (WNV) at necropsy.

Bird #	Weeks post-infection	qRT-PCR C_t_ scores (WN1, WN2)*	Virus isolation
89	3	spln (31.2, 31.4), kid (32.8, 34.7)	
90	3	spln (27.1, 29.1), kid (30.2, 31.6)	
92	3	skin (33.3, UD), spln (24.9, 26.7), kid (26.6, 30.0)	spn, kid
94	3	skin (28.4, 32.7), spln (27.6, 32.9), kid (31.8, 33.8)	
95	3	spln (27.5, 28.7), kid (28.5, 30.5)	spln
97	3	spln (32.1, 34.3), kid (27.5, 30.3)	
98	3	spln (26.4, 28.0), kid (23.3, 25.1), brn (32.6, UD)	
100	3	skin (33.0, UD), spln (30.5, 32.2), kid (27.1, 29.7)	
101	3	spln (28.0, 30.6), kid (25.6, 27.1)	
102	3	spln (29.4, 31.8)	
103	3	spln (24.1, 25.1), kid (23.0, 25.3), brn (32.9, UD)	spln, kid
104	3	spln (28.4, 29.9), kid 28.0, 30.5), brn (30.8, UD)	
105	3	skin(24.4, 28.1), spln (26.6, 27.1), kid (26.3, 28.6)	
504	5	spln (33.5, UD)	
510	5	kid (34.8, UD)	
533	5	spln (29.5, 33.7), kid (35.0, UD)	
553	5	spln (28.9, 28.4), kid (24.7, 26.9)	
564	5	kid (34.1, UD)	
565	5	spln (28.6, 28.8), kid (34.9, UD)	spln
508	7	spln (23.7, 32.4)	
518	7	spln (26.3, UD)	spln
522	7	spln (21.3, 34.3), kid (28.0, 35.1)	spln
527	7	spln (28.8, UD)	
554	7	spln (28.9, UD)	
503	9	skin (35.8, UD)	
520	9	spln (27.4, 33.0), kid (24.9, UD)	
524	9	spln (32.8, UD)	
526	9	skin (33.4, UD)	
548	9	spln 26.5, 30.3)	
61	12	skin (39.7, UD)	
75	12	skin (25.9, 28.9), spln (33.0, 31.4) brn (32.5, 31.0)	spln
76	12	spln (36.8, UD)	
78	12	skin (36.9, UD)	
65	18	kid (27.4, 30.4)	

* spln = spleen, kid = kidney, brn = brain; tissues with positive threshold cycle (C_t_) scores (<40), analyzed with WN1 and WN2 primer/probe sets; UD = C_t_ was undetermined.

Virus isolation was successful, but an infrequent occurrence, with WNV generally isolated from the spleen and on two occasions the kidney (Table 1). Amongst the birds tested for persistence at 3 weeks pi, WNV was isolated from the spleen and kidney of two birds, and the spleen alone from a third. In addition, virus was isolated from the spleen of one bird at 5 weeks pi, two birds at 7 weeks pi, and a final bird at 12 weeks pi. In nearly all cases CPE was observed during the second passage on Vero cells, except for the two spleen samples collected 3 weeks pi, where CPE was detected on the first passage. Despite the addition of amphotericin B to the co-culture medium, skin homogenate cultures were often lost to fungal contamination. This contamination was not seen in other tissues and was attributed to contamination introduced from the skin itself, and not contamination acquired in the laboratory. To prevent contaminating other cultures, skin samples were cultured separately and discarded when/if contamination was noticed. Future attempts may require additional anti-fungal additives to facilitate successful co-culture of skin homogenates.

Biweekly throughout the experiment and at termination, blood samples were collected and sera tested for WNV RNA. Interestingly, ten sparrows had post-acute (≥2 weeks pi) serum samples positive for WNV RNA by qRT-PCR using the WN1 primer/probe set (Table 2). Among these birds, half were positive on more than one sample date, and two were positive up to seven weeks pi. All birds with sera positive for WNV RNA, aside from one (bird #74) that died unexpectedly at three weeks pi, were assessed for persistent infection. Seven of nine birds with WNV RNA positive sera also had tissues that were WNV RNA positive (Table 2). The two birds with negative tissues were necropsied at 15 weeks pi, but had positive sera at two (bird # 550) and two and six (bird #513) weeks pi. Presumably, these birds cleared their persistent infections prior to assessment at 15 weeks pi. Based on estimates from our qRT-PCR standards (Figure 3), C_t_ scores obtained from WNV RNA positive sera corresponded to very low viral titers of approximately 10–100 pfu/mL.

**Table 2:** House Sparrows (*Passer domesticus*) with serum positive for WNV RNA beyond the acute infection period ≥2 weeks post-infection.

Bird #	wpi^a^	Total time held (wpi)	qRT-PCR positive tissue^b^	Co-culture positive tissue
74	2	3	NT	−
75	4	12	+	+
508	4,7	7	+	−
513	2, 6	15	−	−
520	2, 4, 6	9	+	−
524	2, 4, 6	9	+	−
527	7	7	+	−
548	2, 4, 6	9	+	−
550	2	15	−	−
553	5	5	+	−

a wpi = weeks post-infection.

b for details on which tissue were positive see Table 1, bird 74 died unexpectedly and was not tested (NT) for persistent infection.

As reported above, 11 experimentally infected birds failed to show a viremia response by plaque assay at 4 dpi, although sera from these 11 birds were positive for WNV RNA by qRT-PCR using WN1. Viral RNA in 10 of these 11 birds was confirmed by qRT-PCR using WN2. The WN1 C_t_ scores from positive samples ranged from 28.7 to 32.9; the sample that failed to confirm had a WN1 C_t_ score of 35.1. In contrast, of the 18 post-acute serum samples that were qRT-PCR positive with WN1, only one sample (bird 74, two weeks pi) was confirmed by WN2, and this sample had a WN1 C_t_ score of 27.1; the remaining 17 positive sera had a mean (± standard deviation) WN1 C_t_ score of 35.6±1.02. The un-confirmed samples were re-extracted and qRT-PCR repeated using WN1; 35% of these samples were confirmed by re-extraction. Repeated freeze-thaw of samples that contained very low copy numbers of target RNA may have reduced the number of samples that re-confirmed by this method.

## Discussion

WNV-infected House Sparrows, and other bird species, develop persistent WNV infections. In House Sparrows these persistent infections were increasingly undetectable with time post-infection and existed concurrently with elevated serum antibody titers. In the current study, WNV RNA was detected in the kidney of one bird at 18 weeks pi and infectious virus was isolated from the spleen of another bird at 12 weeks pi. The isolation of infectious virus indicated the persistence of intact WNV and not just residual RNA from the acute infection.

Experimental infection was conducted with an isolate of the North American or WN02 genotype of WNV circulating in California during 2004, which differed from previous studies of WNV persistence that utilized NY99 isolates [13], [15], [17] or infectious clones [14]. However, our previous study [18] reported that the infecting WNV strain did not significantly alter the proportion of birds that developed persistent infection. Both this study and research published previously by Nemeth et al. [17], in which birds were infected with NY99, reported similar proportions of House Sparrows positive for persistent WNV (WNV RNA from any tissue) at approximately 9 weeks pi. Here, 5 of 10 House Sparrows were positive for persistence, whereas Nemeth et al. [17] reported 2 of 14 positive. Although sample sizes were low, there was no significant difference in these two proportions (Fisher's exact; *P*>0.05).

The mean 4 dpi viremia of experimentally infected birds in the current study was 3.5 log_10_ pfu/mL of sera, and was much lower than previous WNV experimental infection studies in House Sparrows that reported 5.0 [4], 9.9 [27] and 10.3 log_10_ pfu/mL [3] at 4 dpi. This decrease in amplitude of the 4 dpi viremia is interesting and has important implications for the transmission of WNV, because decreases in viremia titer are well correlated with decreases in host competence or the proportion of mosquitoes infected through blood-feeding [28]. Although, it is not specifically known why the House Sparrows in our study developed a lower 4 dpi viremia, nearly eight years of WNV selection pressure on House Sparrows from the collection areas may have led to selection for birds with innate resistance. In addition, birds in our study may have been under less sampling stress, because they were only handled and bled once during the acute infection period. To improve overall colony health, sparrows were treated with chlorotetracycline prior to WNV infection. Although this antibiotic may have improved the health status of the birds, chlorotetraclcline has been demonstrated to be ineffective against WNV [29] and should not have altered the course of infection. Regardless of the cause of the decreased viremic response, this outcome did not appear to impact persistence.

Ten experimentally infected birds had sera that were positive for WNV RNA from 2 to 7 weeks pi and after the acute infection period. It was not clear whether sera were intermittently positive, due to recrudescence, or if some birds simply maintained low-grade persistent viremias. Three birds (520, 524, and 548) appeared to have developed persistent viremias, and two birds (508 and 513) alternated positive/negative/positive. The five remaining birds were RNA-positive on only one occasion. Quantities of RNA detected corresponded to extremely low viral titers of 10–100 pfu/mL of serum. Blood was drawn in 0.1 mL volumes that were diluted to create a 1∶10 serum dilution. Because of the small collection volume and dilution, it is possible that some samples containing 10–100 pfu/mL may have been missed through sampling. Confirmation of these low-grade viremias was vexing; however, several birds were positive on more than one occasion, negative controls were always negative, and all but two birds tested 15 weeks post-infection had one or more tissues positive for WNV RNA at necropsy. Despite the fact that some birds developed either low-grade persistent or recrudescent viremias, these viremias would not likely result in mosquito infection. We previously demonstrated that avian antibodies protect mosquitoes from WNV infection [28], and all birds in the current study retained robust antibody titers sufficient to bind virus at these low viremia levels.

Experimentally infected House Sparrows developed and maintained robust neutralizing antibody titers post-infection. Previous studies reported this response in both House Sparrows [26] and mice [14]. It is becoming apparent that WNV infections are not always cleared after acute infection. These lingering or perhaps recrudescent infections may provide continual stimulation to the host immune system, thus serving to amplify and maintain the humoral response. However, similar to our previous study [18], antibody titers at necropsy of persistently infected birds in the current study were not significantly higher than the titers of birds negative for persistence. These analyses may have been confounded by the loss of detectable RNA over time, so that some of the birds, such as those that had post-acute serum positive for WNV RNA (birds 513 and 550) but tissues negative at necropsy, may have cleared persistent infections prior to sampling.

At this time, events or conditions that may initiate a recrudescence event and thus restart transmission in nature have not been elucidated. Optimizing the limit of virus detection is critical for characterization of WNV persistence and therefore some birds were reported as qRT-PCR positive, even if they failed to confirm via WN2 or re-extraction and WN1 testing. Although a confirmatory primer/probe set can be useful for avoiding a type I error, results may be confounded if the primer/probe used is less sensitive than the screening probe. In contrast, a type II error would certainly result if all samples that failed to confirm were disregarded. To balance these two types of error, extreme care was used to avoid cross-contamination, numerous negative controls were included on RNA extraction and qRT-PCR plates, and samples with high C_t_ scores were re-extracted and qRT-PCR repeated.

Our study confirmed previous reports of WNV persistence in avian hosts [13], [16]–[18], and further characterized the dynamic nature of these infections utilizing a House Sparrow model. Persistent infections appeared to resolve steadily with increasing time post-infection. Although, virus isolation was not a common occurrence, recovery of infectious virus at 12 weeks pi indicated that persisting RNA may be attributed to infectious, intact virus, but was likely moderated by the host immune system. West Nile virus RNA was detected in the sera of persistently infected birds up to 7 weeks pi, but it was not clear if these low-grade post-acute viremias were persistent or recrudescent.

All experimentally infected birds maintained neutralizing antibody titers throughout the experiment. When present in sufficient quantity, host neutralizing antibodies, consumed within a bloodmeal, protected mosquito vectors from WNV infection [28]. Therefore, it currently appears that healthy birds that maintain neutralizing antibody titers are unlikely to develop a mosquito-infectious recrudescent viremia. However, evidence indicates that persistent infections maintain virulence. Therefore, factors that compromise a persistently infected bird's immune system possibly could enable mosquito-infectious recrudescent viremias. In some cases, WNV infections in avian hosts persisted sufficiently long to serve as a potential overwintering mechanism for the virus. However, for proof-of-principle persistent WNV infections must reinitiate a transmission event either through a mosquito-infectious viremia or bird to bird transmission. Future work is required to determine which factors enable recrudescence and whether this occurs in nature.
